# Social connectedness as a predictor of suicidal outcomes in psychiatric outpatients: A 12-month prospective EMA study

**DOI:** 10.1192/j.eurpsy.2026.11342

**Published:** 2026-07-31

**Authors:** A. A. Porras-Segovia, F. M. Herrmann, L. Albarracín-García, C. Díaz-Téllez, E. Baca-García

**Affiliations:** 1 Translational Psychiatry, Health Research Institute Fundación Jiménez Díaz, Madrid, Spain; 2 Psychology, Harvard University, Boston, United States; 3 Hospital Clinic, Barcelona, Spain

## Abstract

**Introduction:**

Suicide is a leading global public health problem, accounting for over 700,000 deaths each year. Beyond its lethality, suicidal thoughts and behaviours carry a significant morbidity burden. Social connectedness has been identified as a key protective factor for suicide, yet most studies rely on static, cross-sectional assessments. Ecological Momentary Assessment (EMA) allows for the capture of real-time fluctuations in risk factors and the protective nature of social connectedness.

**Objectives:**

This study aims to advance the field by examining the prospective association between EMA-measured social connectedness and suicidal outcomes (both EMA-based and EHR outcomes), over a 12-month EMA follow-up period.

**Methods:**

We conducted a 12-month prospective EMA study involving 550 psychiatric outpatients from four Spanish hospitals who had experienced recent suicidal thoughts or attempts. Participants received a daily survey containing 2–4 randomly selected items from a pool of 34. These items included measures of social connectedness, active and passive suicidal ideation, and non-suicidal self-injury (NSSI). Information on suicide attempts was extracted from electronic medical records. Multilevel mixed-effects models were used to distinguish between-person (average levels) and within-person (daily deviations) associations, adjusting for age, sex, time, and missingness.

**Results:**

Across 81,023 completed assessments, higher average connectedness was significantly associated with lower active suicidal ideation (b = −0.18, p = .014) and NSSI (b = −0.28, p = .003). Within-person increases in connectedness also predicted reductions in active suicidal ideation (b = −0.12, p < .001), but not NSSI or passive ideation. Lastly, the only significant predictor of suicide attempts was the missingness of active suicidal ideation EMA in the 7 days prior.

**Table 1.** Descriptive and variability statistics for suicide outcomes and social connectedness Table 1. Long description.

	Descriptive statistics				Variability statistics	
Variable	M	SD	Range	% nonzero	ICC	RMSSD
Active suicidal ideation	50.096	34.144	0–100	93	0.079	36.785
Passive suicidal ideation	44.856	33.243	0–100	89.9	0.106	39.416
Non-suicidal self-injury	14.464	25.823	0–100	51.5	0.243	21.458
Social connectedness	49.357	33.586	0–100	91	0.18	37.236

*Note.* M = mean; SD = standard deviation; % nonzero = proportion of responses greater than zero; ICC = intraclass correlation coefficient, indexing the proportion of variance attributable to between-person differences; RMSSD = root mean square of successive differences.

**Conclusions:**

Social connectedness predicts active suicidal ideation at both the between- and within-person levels. Lower connectedness is also associated with a greater risk of NSSI. Long-term EMA offers valuable insights suggesting that connectedness should be incorporated into suicide monitoring and intervention strategies.

**Disclosure of Interest:**

None Declared

## Long descriptions

### Table 1. Long description

The table is organized into seven columns. The first column lists the variables, followed by four columns under the heading Descriptive statistics (M, S D, Range, and percent nonzero) and two columns under Variability statistics (I C C and R M S S D).

* Active suicidal ideation: M 50.096, S D 34.144, Range 0 to 100, 93 percent nonzero, I C C 0.079, R M S S D 36.785.

* Passive suicidal ideation: M 44.856, S D 33.243, Range 0 to 100, 89.9 percent nonzero, I C C 0.106, R M S S D 39.416.

* Non-suicidal self-injury: M 14.464, S D 25.823, Range 0 to 100, 51.5 percent nonzero, I C C 0.243, R M S S D 21.458.

* Social connectedness: M 49.357, S D 33.586, Range 0 to 100, 91 percent nonzero, I C C 0.18, R M S S D 37.236.

Note: M equals mean; S D equals standard deviation; percent nonzero equals proportion of responses greater than zero; I C C equals intraclass correlation coefficient; R M S S D equals root mean square of successive differences.

Navigate back to Table 1..

